# Induction of ER Stress in Acute Lymphoblastic Leukemia Cells by the Deubiquitinase Inhibitor VLX1570

**DOI:** 10.3390/ijms21134757

**Published:** 2020-07-04

**Authors:** Paola Pellegrini, Karthik Selvaraju, Elena Faustini, Arjan Mofers, Xiaonan Zhang, Jens Ternerot, Alice Schubert, Stig Linder, Pádraig D′Arcy

**Affiliations:** 1 Department of Biomedical and Clinical Sciences, Linköping University, S-58183 Linköping, Sweden; paola.pellegrini@vlvbio.com (P.P.); karthik.selvaraju@liu.se (K.S.); elena.faustini@liu.se (E.F.); arjan.mofers@liu.se (A.M.); jente480@student.liu.se (J.T.); alisc838@student.liu.se (A.S.); stig.linder@ki.se (S.L.); 2 Department of Immunology, Genetics and Pathology, Uppsala University, 751 85 Uppsala, Sweden; xiaonan.zhang@igp.uu.se; 3 Department of Oncology-Pathology, Karolinska Institute, S-17176 Stockholm, Sweden

**Keywords:** acute lymphocytic leukemia, proteasome, translation, bortezomib

## Abstract

The proteasome is a validated target of cancer therapeutics. Inhibition of proteasome activity results in the activation of the unfolded protein response (UPR) characterized by phosphorylation of eukaryotic initiation factor 2α (eIF2α), global translational arrest, and increased expression of the proapoptotic CHOP (C/EBP homologous protein) protein. Defects in the UPR response has been reported to result in altered sensitivity of tumor cells to proteasome inhibitors. Here, we characterized the effects of the deubiquitinase (DUB) inhibitor VLX1570 on protein homeostasis, both at the level of the UPR and on protein translation, in acute lymphoblastic leukemia (ALL). Similar to the 20S inhibitor bortezomib, VLX1570 induced accumulation of polyubiquitinated proteins and increased expression of the chaperone Grp78/Bip in ALL cells. Both compounds induced cleavage of PARP (Poly (ADP-ribose) polymerase) in ALL cells, consistent with induction of apoptosis. However, and in contrast to bortezomib, VLX1570 treatment resulted in limited induction of the proapoptotic CHOP protein. Translational inhibition was observed by both bortezomib and VLX1570. We report that in distinction to bortezomib, suppression of translation by VXL1570 occurred at the level of elongation. Increased levels of Hsc70/Hsp70 proteins were observed on polysomes following exposure to VLX1570, possibly suggesting defects in nascent protein folding. Our findings demonstrate apoptosis induction in ALL cells that appears to be uncoupled from CHOP induction, and show that VLX1570 suppresses protein translation by a mechanism distinct from that of bortezomib.

## 1. Introduction

Approximately 85% of pediatric leukemias are comprised of acute lymphoblastic leukemia [1]. The most common forms are of B-cell lineage, and the remaining are of T cell lineage [2]. The disease is managed by an initial phase of induction therapy based on vincristine and dexamethasone, usually followed by treatment with methotrexate and cytarabine [3]. Postremission consolidation is most often followed by long-term maintenance with mercaptopurine and methotrexate. Treatment is generally successful with survival rates on the order of 80%, but ALL treatments still require improvement. A particular concern is the risk of secondary cancers due to exposure to genotoxic agents early in life [4].

In cancer cells, the accumulation of genetic mutations, aneuploidy, and chromosomal alterations, coupled with a high proliferative index, result in increased protein synthesis and a “proteostasis” addiction to pathways controlling homeostasis, heat shock, and the unfolded protein response (UPR) [5,6]. This dependence on mechanisms controlling protein homeostasis has been shown to lead to sensitivity to defects in the ubiquitin proteasome pathway and to inhibitors such as bortezomib and carfilzomib [7]. Among the different mechanisms that have been described underlying the cytotoxicity of proteasome inhibitors to tumor cells, induction of endoplasmic reticulum (ER) stress and the unfolded protein response (UPR) is generally accepted to play a major role [8]. An ER stress-related mechanism of action of proteasome inhibitors is consistent with the sensitivity of multiple myeloma cells, characterized by high levels of immunoglobulin synthesis, to proteasome inhibition [9].

Phosphorylation of eukaryotic initiation factor 2α (eIF2α) at serine-51, inhibiting nucleotide exchange by eukaryotic translation initiation factor 2 subunit beta (eIF2B), is a central component of the UPR. Different protein kinases have been shown to induce of eIF2α phosphorylation, the ER stress-activated PERK (protein kinase R (PKR)-like endoplasmic reticulum kinase) and the heat shock-activated HRI (heme-related eIF2α kinase) being most relevant in the present context [10]. eIF2α phosphorylation converts eIF2-GDP into a competitive inhibitor of eIF2B, thereby slowing protein synthesis, while at the same time inducing the production of a set of transcription factors, including Activating Transcription Factor 4 (ATF4) [11,12]. The cytoplasmic levels of eIF2B are ~10–20% that of eIF2α, thus phosphorylation of as little as 10% of eIF2α can be sufficient to sequester virtually all the available eIF2B [13,14]. Translation of the transcription factor ATF4 is promoted during conditions of eIF2α phosphorylation, leading to increased expression of the transcription factor CHOP (C/EBP homologous protein; also referred to as DDIT3 and GADD153) [15]. In some cell lines, the strong cytotoxicity of the 20S proteasome inhibitor bortezomib has been speculated to be coupled to a lack of eIF2α phosphorylation, leading to a loss of a protective response [16,17,18]. The amplitude of eIF2α phosphorylation in response to bortezomib has been found to affect cellular sensitivity to bortezomib [17,19,20]. For a recent review on the integrated stress response and its role in determining sensitivity and resistance to proteasome inhibitors, see [10].

Proteasome activity can be blocked at the level of the 19S proteasome [21,22]. A number of compounds have been described to target the 19S proteasome by inhibiting the cysteine deubiquitinases (DUBs) USP14/UCHL5 [21], the metalloprotease DUB POH1 [23], or the Rpn13 receptor [24]. Inhibitors of proteasome associated deubiquitinases by compounds such as b-AP15, VLX1570, RA-9, and G5 induce cell death by mechanisms that appear to be different from 20S proteasome inhibitors such as bortezomib, possibly due to additional targets [21,25,26,27]. We and others have shown that b-AP15/VLX1570 induce ER stress and induction of eIF2α phosphorylation in colon carcinoma [28], melanoma [29], Waldenströms macroglobulinaemia [30], prostate carcinoma [31], and hepatocellular carcinoma cells [32]. However, we have also reported that treatment of ALL cells with the proteasome DUB inhibitor VLX1570 did not induce detectable phosphorylation of eIF2α, whereas protein synthesis was nevertheless inhibited [33]. Whether this phenomenon also applies to the clinically used proteasome inhibitor bortezomib and whether ALL cells have a defect in the UPR is unclear. Here, we examined this question and found that bortezomib also induces low levels of eIF2α phosphorylation in ALL cells, but nevertheless induces CHOP expression. VLX1570 elicits weak induction of CHOP, but is capable of inducing apoptosis in ALL cells. We also report that VLX1570 inhibits protein synthesis at the level of translational elongation.

## 2. Results

### 2.1. The DUB Inhibitor VLX1570 Elicits ER Stress in ALL Cell Lines, but only Weak Induction of CHOP

**Figure 1:**
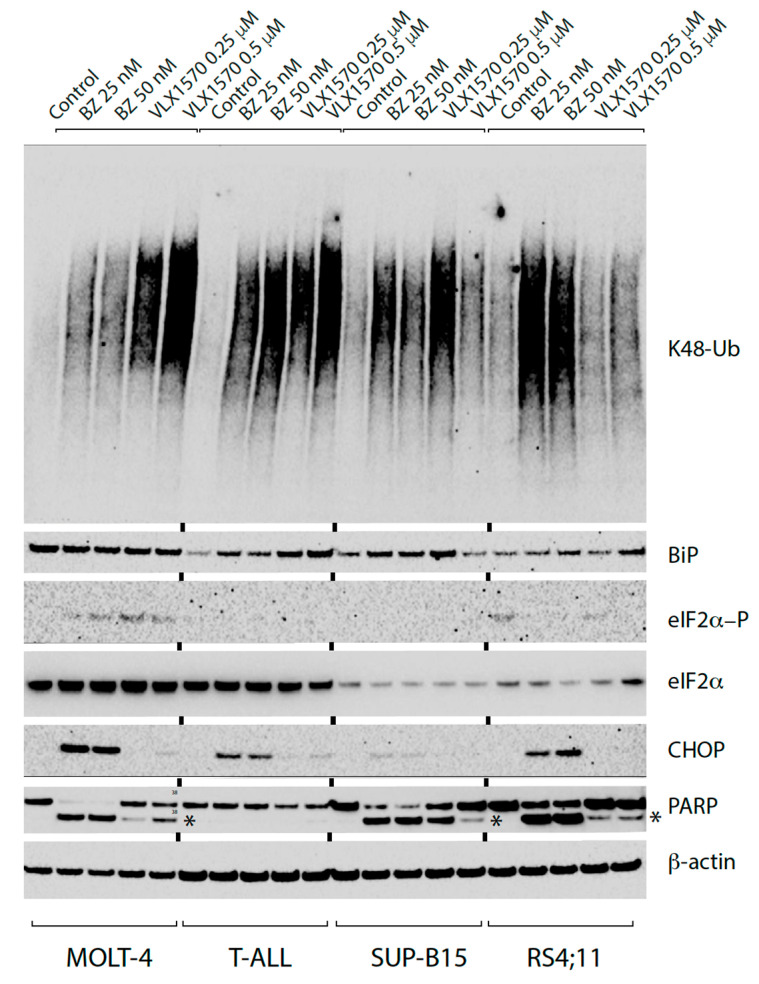
Accumulation of polyubiquitinated proteins and induction of endoplasmic reticulum (ER) stress in acute lymphoblastic leukemia (ALL) cells exposed to bortezomib or VLX1570. T-ALL (MOLT-4 and T-ALL) and B-ALL (SUP-B15 and RS4;11) cells were exposed to bortezomib (BZ), VLX1570 or vehicle (0.5% DMSO; control) for 9 h, and extracts were prepared and subjected to immunoblotting using the indicated antibodies. The antibody to polyubiquitin detects K48-linked chains; Poly (ADP-ribose) polymerase (PARP) cleavage fragments are indicated with an asterix.

The response to two inhibitors of the UPS, the 20S proteasome inhibitor bortezomib (BZ) and the DUB inhibitor VLX1570, was examined in four ALL cell lines. These cell lines were chosen from a panel of B-ALL and T-ALL cell lines previously shown to be sensitive to VLX1570 [33]. Both drugs induced the accumulation of K48-linked polyubiquitin chains in ALL cells after 6 and 9 h of drug exposure (Figure 1, Figure S1). Proteasome inhibitors have been demonstrated to elicit ER stress and we observed increased expression of the ER chaperone Grp78/BiP in the two B-ALL cell lines SUP-B15 and RS4;11. Grp78/BiP was also induced in T-ALL cells, but not in MOLT-4 cells, possibly due to high levels of basal expression (Figure 1). ER stress is associated with induction of the integrated stress response (ISR) [34], resulting in phosphorylation of eIF2α and inhibition of protein synthesis. Phosphorylation of eIF2α was observed in MOLT-4 cells exposed to VLX1570, but was generally weak (Figure 1). The low levels of induction of eIF2α phosphorylation in SUP-B15 and RS4;11 cells may be explained by the low levels of eIF2α in these cells (Figure 1, Figure S2).

Phosphorylation of eIF2α results in repression of translation initiation [35]. Some mRNAs, including *CHOP* (DDIT3/GADD153) mRNA, are however translated [36]. *CHOP* expression is induced at the transcriptional level and CHOP protein levels are regarded as a measure of sustained ER stress and have been linked to apoptosis [37]. Bortezomib was found to induce CHOP expression in three out of four ALL cell lines (Figure 1). The fourth cell line, SUP-B15, was not generally unresponsive to induction of CHOP since the thapsigargin, an inhibitor of the sarco/endoplasmic reticulum calcium ATPase, induced CHOP in these cells (Figure S3).

**Figure 2:**
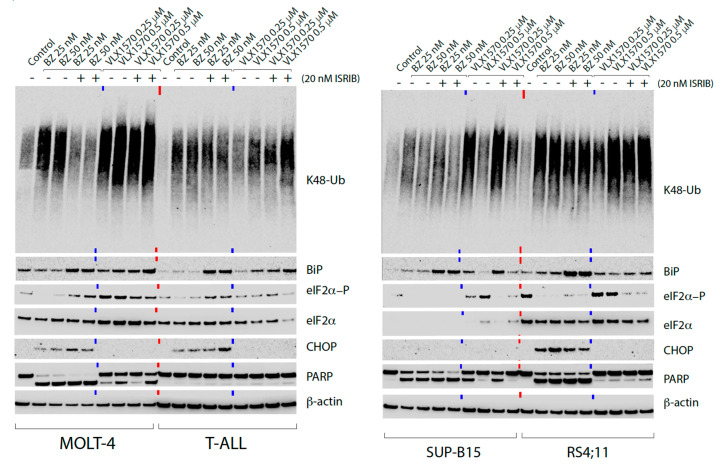
Effect of Integrated Stress Response Inhibitor (ISRIB) on endoplasmic reticulum (ER) stress induced by bortezomib and VLX1570. Cells were exposed to bortezomib, VLX1570, or vehicle (0.5% DMSO) for 9 h. Extracts were prepared and subjected to immunoblotting using the indicated antibodies.

In contrast to bortezomib, VLX1570 induced weak or no detectable CHOP in the ALL cell lines (Figure 1 and Figure 2). This finding raised the possibility that apoptotic signaling was not induced by VLX1570. We did, however, find induction of PARP cleavage by both VLX1570 and bortezomib in three out of four cell lines at 9 h of exposure (Figure 1). In the remaining cell line, T-ALL, PARP cleavage was observed at 12 h (Figure S4). We conclude from these experiments that both types of UPS inhibitors induce ER stress in ALL cells, but that the responses differ. The 20S proteasome inhibitor bortezomib induces low or sometimes undetectable levels of eIF2α phosphorylation but nevertheless induced CHOP expression in three of four cell lines. In contrast, VLX1570 induces weak or no detectable CHOP expression despite a generally stronger stimulation of eIF2α phosphorylation (Figure 1 and Figure 2).

### 2.2. The eIF2B Activator ISRIB Enhances Induction of BiP

The apparently low induction of eIF2α phosphorylation is difficult to interpret in mechanistic terms since low levels of phosphorylated eIF2α may be sufficient to sequester all available eIF2B [13,14]. We used the small molecule ISRIB (Integrated Stress Response Inhibitor), an activator of eIF2B [38], to examine the potential role of eIF2α signaling in the response to bortezomib and VLX1570 in ALL cells. ISRIB increased the induction of Grp78/BiP by bortezomib in all four cell lines and by VLX1570, although less consistently so (Figure 2). This result suggests a role of phosphorylated eIF2α to constrain ER stress, resulting in an increase of Grp78/BiP in ISRIB-exposed cells. eIF2α phosphorylation was more variably affected. ISRIB enhanced eIF2α phosphorylation by bortezomib in MOLT-4 and T-ALL cells, consistent with the release of a feedback mechanism. Cotreatment with ISRIB generally decreased eIF2α phosphorylation in cells exposed to VLX1570 (Figure 2). ISRIB had minor effects on CHOP induction in cells treated with bortezomib and did not enhance CHOP expression by VLX1570 (Figure 2).

### 2.3. Bortezomib and VLX1570 Inhibit Translation

**Figure 3:**
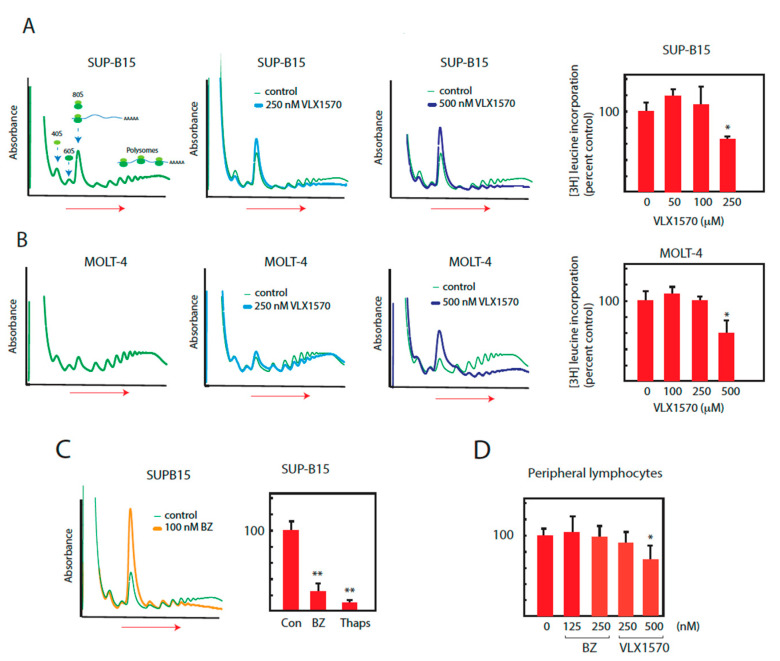
Suppression of translation by bortezomib and VLX1570. Cells were exposed to bortezomib or VLX1570, and ribosomes and polysomes were fractionated using sucrose gradient centrifugation. Incorporation of [^3^H]-leucine into acid precipitable material was measured after 6 h of drug exposure. (**A,B**) SUP-B15 or MOLT-4 cells were exposed to VLX1570 for 6 h and lysates were prepared in the presence of RNAase inhibitors and cycloheximide (100 μg/mL) and fractionated by sucrose gradient sedimentation (left to right). Absorbance (A_280_) was monitored during collection. The dose response of [^3^H]-leucine incorporation into acid precipitable material was determined after 6 h of drug exposure ([^3^H]-leucine was added during the last hour). Mean values ± S.D., * *p* < 0.05 Student′s *t*-test. (**C**) SUP-B15 cells were exposed to bortezomib (BZ) and lysates were fractionated by sucrose gradient sedimentation (left to right) and A_280_ monitored during collection. [^3^H]-leucine incorporation into acid precipitable material was measured after 6 h exposure to 100 nM bortezomib or 10 µM thapsigargin. Mean values + S.D., ** *p* < 0.01, Student′s *t*-test. (**D**) Peripheral lymphocytes were exposed to VLX1570 for 6 h and [^3^H]-leucine incorporation into acid precipitable material was determined. Mean values ± S.D., * *p* < 0.05, Student′s *t*-test.

The ISR is generally considered to be a protective mechanism, leading to decreased translation and hence decreased production of misfolded proteasome substrates. Our inability to detect phosphorylation of eIF2α in SUP-B15 cells exposed to bortezomib or VLX1570 raised the possibility that the lack of ISR results in elevated sensitivity to these compounds. SUP-B15 was previously found to be sensitive to VLX1570 using proliferation (MTT) assays [33], more so than MOLT-4 cells (IC_50_: 50 ± 15 nM for SUP-B15 and 152 ± 24 nM for MOLT-4 cells). In order to study the effects of drugs on translation, we examined polysome profiles. Cycloheximide was used to arrest elongating ribosomes, and lysates were subjected to sucrose gradient ultracentrifugation [39]. VLX1570 elicited a decrease in the number of mRNAs associated with large polysomes and an increase in 80S ribosomes in both MOLT-4 cells and SUP-B15 cells (Figure 3A,B), showing an overall decrease in translating ribosomes. In MOLT4 cells, this effect was observed at 500 nM VLX1570 and in SUP-B15 cells at 250 nM. The higher sensitivity of SUP-B15 cells to protein synthesis inhibition was also confirmed in experiments where incorporation of [^3^H]-leucine into acid precipitable material was determined as a measure of global protein synthesis (Figure 3A,B).

Despite the lack of detection of eIF2α phosphorylation in SUP-B15 cells exposed to bortezomib, decreases in large polysomes and increases in monosomes, as well as decreases in [^3^H]-leucine incorporation, were observed in bortezomib-treated SUP-B15 cells (Figure 3C). Decreases in [^3^H]-leucine incorporation were also found in response to thapsigargin, known to induce ER stress and the ISR (Figure 3C). We finally examined whether inhibition of protein translation is observed in lymphocytes, previously found to show limited sensitivities to proteasome DUB inhibitors [21,40]. We found that peripheral lymphocytes showed a similar response as MOLT-4 cells to VLX1570 with suppression of incorporation of [^3^H]-leucine observed at 500 nM (Figure 3D).

### 2.4. Limited Effects of the eIF2B Activator ISRIB on Translational Inhibition by VLX1570

**Figure 4:**
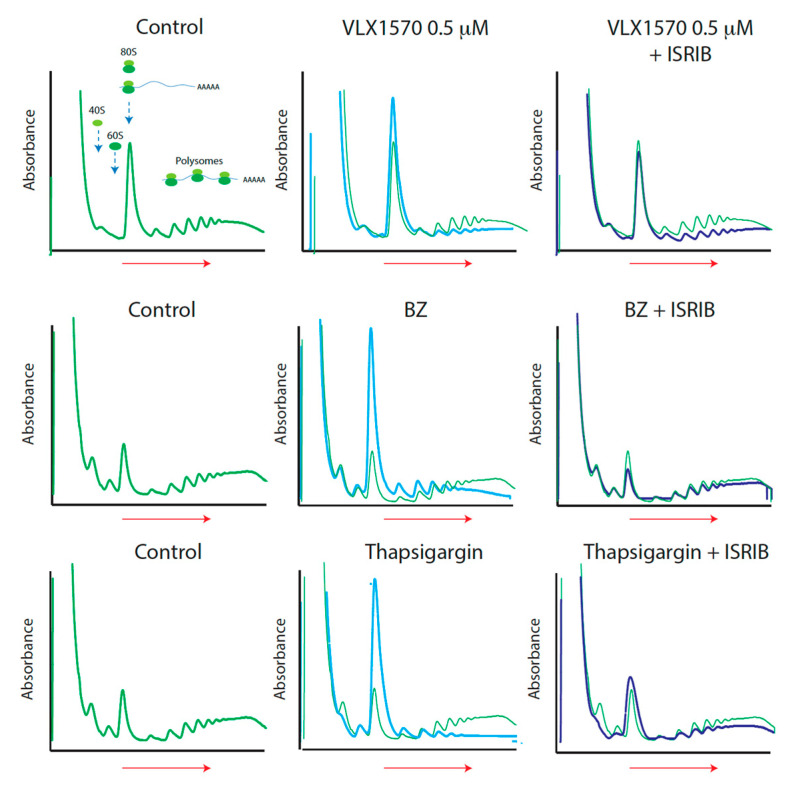
Different sensitivities of drug-suppressed translation to the eukaryotic translation initiation factor 2 subunit beta (eIF2B) activator integrated stress response inhibitor (ISRIB). Cells were exposed to 0.5 µM VLX1570, 100 nM bortezomib, or 10 μM thapsigargin in the presence or absence of 0.2 μM ISRIB for 6 h. Lysates were prepared as subjected to sucrose gradient sedimentation (left to right). Absorbance (A_280_) was monitored during collection.

ISRIB will override the negative effect of phosphorylated eIF2α to stimulate translation [38]. The strong increases in 80S peaks and decreases of polysomes observed in bortezomib- and thapsigargin-exposed cells were reversed by ISRIB (Figure 4). In contrast, ISRIB treatment led to only minor effects on the levels of polysomes in VLX1570-exposed cells (Figure 4). This finding suggests that the decreases in mRNA-associated polysomes observed in VLX1570-treated cells were not due to inhibition of translation at the level of eIF2α-P/eIF2B.

A compound related to VLX1570, a biotin-conjugated compound named 2c, was previously shown to bind to AKT (protein kinase B) [26]. We therefore considered the possibility that decreased protein synthesis in VLX1570-exposed cells could be explained by decreased mammalian target of rapamycin (mTOR) signaling. However, increased phosphorylation of mTOR was observed in response to VLX1570 (Figure S5).

### 2.5. Evidence for Elongational Block by VLX1570 Treatment

**Figure 5:**
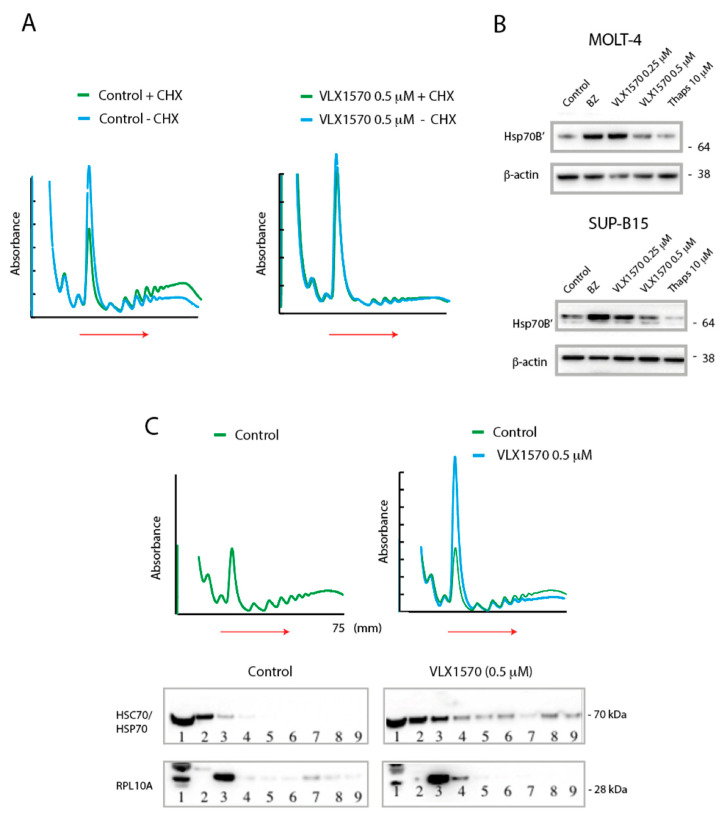
Evidence for impaired translational elongation. (**A**) SUP-B15 cells were exposed to VLX1570 or vehicle and extracts fractionated by sucrose gradient centrifugation. Extracts were prepared in the presence or absence of cycloheximide (CHX) as indicated. (**B**) MOLT-4 or SUP-B15 cells were exposed to the indicated compounds for 6 h, and cell extracts were processed for immunoblotting. Bortezomib (BZ) was used at 50 nM. (**C**) SUP-B15 cells were exposed to VLX1570 or vehicle and extracts fractionated by sucrose gradient centrifugation. Fractions were collected and analyzed by immunoblotting using antibodies to Rpl10A (a component of the 60S ribosome subunit) or HSC70/HSP70 proteins.

The standard protocol for performing polysome profiling includes the use of cycloheximide to stall ribosomes on transcripts during preparation of lysates. Omission of cycloheximide leads to ribosome “run-off” and decreases in the polysome fraction [39]. The omission of cycloheximide from control cell lysates resulted in the expected decrease in polysomes (Figure 5A). In contrast, omission of cycloheximide during the preparation of lysates from VLX1570-treated cells had no detectable effect on polysome profiles (Figure 5A).

Proteasome deubiquitinase inhibitors induce high levels of expression of Hsp70 mRNA transcripts, particularly the highly inducible isoform HSPA6 [27,28,41,42]. We found that Hsp70B′ (encoded by the HSPA6 gene) did not increase in a dose-dependent manner in MOLT-4 and SUP-B15 cells (Figure 5B). Hsp70 transcripts contain internal ribosome entry site that allows cap-independent translation during stress conditions [43], and the response to VLX1570 is consistent with inhibition of elongation.

We considered the possibility that translation elongation may be inhibited due to functional depletion of Hsp70 proteins, a mechanism described to occur during severe heat shock [44]. In this scenario Hsp70 proteins become increasingly associated with misfolded proteins accumulating in cells, leading to deficiency at the level of translating ribosomes and inhibition of elongation [44]. Surprisingly, however, we observed increased levels of Hsp70 proteins in the polysome fractions from VLX1570-exposed cells (Figure 5C).

### 2.6. VLX1570 Reduces the Proliferation of RS4:11 Cells in Zebrafish Embryos

**Figure 6:**
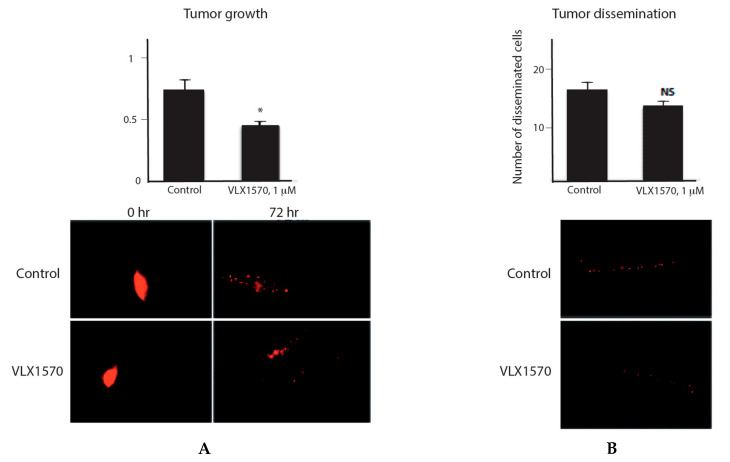
Antineoplastic activity in the zebrafish embryo model. (**A, left**) Assessment of tumor cell growth in zebrafish embryos. Embryos (*n* = 20) were injected with labeled ALL cells and fluorescence determined after injection (basal level) and after 72 h (average ± S.E.M.). (**B, right**) Assessment of tumor dissemination in zebrafish embryos. Embryos (*n* = 20) were injected with labeled ALL cells and labeled cells in dorsal regions were recorded after 72 h (average ± S.E.M.). Statistical significance was calculated by *t*-test; * *p* < 0.05. Scale bar 200 µm

VLX1570 has passed GLP toxicity protocols and entered into clinical studies [45]. In vivo use requires the use of oil/detergents for formulation, leading to complications in the evaluation of toxicology due to species differences in the tolerance to such formulations [46]. A Phase 1 study using VLX1570 in a PCT formulation (PEG/Chremophore/Tween; polyethylene glycol, polyoxyethylated castor oil and polysorbate 80) encountered pulmonary toxicity [45]. Pulmonary toxicity has, however, been observed also using bortezomib [47], and the PCT formulation enhances this type of toxicity for bortezomib [45]. In order to examine toxicity and activity of VLX1570 to ALL cells, we used the zebrafish model. This model has been advocated as a suitable model for toxicity assessment of anticancer compounds [48]. Zebrafish development (0–120 h) was not affected by the addition of 1 µM VLX1570 to the water. This concentration did, however, significantly reduce the proliferation of RS4:11 cells in the embryos (Figure 6).

## 3. Discussion

Here, we report that the DUB inhibitor VLX1570 induces ER stress in ALL cells, but induces only weak increases of CHOP. The difference with regard to CHOP induction by VLX1570 and bortezomib could not be explained by lower levels of phosphorylation of eIF2α by VLX1570, and the mechanism underlying this defective response is not understood at present. Where examined, VLX1570 induced inhibition of translational elongation, and it is possible that CHOP translation is affected. However, we find this less likely since the increases of Hsp70B′ were similar in MOLT-4 cells exposed to 0.25 μM VLX1570 and 50 nM bortezomib; suppression of Hsp70B′ induction was most evident in SUP-B15 cells at 500 nM VLX1570 (Figure 5B). The findings suggest that VLX1570 induces apoptotic signaling in ALL cells by mechanisms independent of the eIF2α–CHOP axis and are consistent with previous reports suggesting proteotoxicity and organelle stress as important for apoptosis/cell death [27].

The sensitivity of SUP-B15 cells to VLX1570 and bortezomib is higher than that of MOLT-4 cells. We initially hypothesized that the sensitivity of SUP-B15 cells to apoptosis/cell death induction by VLX1570 could be attributed to the limited induction of eIF2α phosphorylation, resulting in a lack of repression of protein synthesis and a defective protective response. However, despite low/undetectable levels of phosphorylated eIF2α in response to VLX1570 and bortezomib, translation was nevertheless suppressed in SUP-B15 cells. The relatively high level of sensitivity does, therefore, not seem to be due to a defective UPR. Translation repression by bortezomib in SUP-B15 cells could be reversed by the eIF2B activator ISRIB. As discussed above, intracellular levels of eIF2B are low compared to eIF2α, and phosphorylation of small amounts of eIF2α can be sufficient to sequester virtually all the available eIF2B [13,14]. This observation raises caution with regard to the utility of eIF2α phosphorylation as a biomarker for sensitivity to proteasome inhibitors in ALL cells, in distinction to what has been demonstrated for pancreas cancer cells [17].

In contrast to bortezomib and thapsigargin, translational suppression by VLX1570 was only marginally alleviated by ISRIB. Furthermore, no increases in 80S peaks or decreases in the polysome fraction were observed after omitting cycloheximide from the cell lysis buffer. These observations are consistent with a block at the level of elongation. It was previously reported that heat shock may lead to inhibition of translational elongation due to depletion of chaperones necessary for folding of nascent chains [44]. During severe heat shock, an increase in light polysomes was observed, consistent with translational pausing after translation of approximately 65 amino acids [44]. This pattern is distinct from that observed during treatment with VXL1570, where decreases in heavy polysomes were preferentially observed. We nevertheless considered the possibility of functional chaperone depletion considering previous findings of proteasome deubiquitinase inhibitors inducing high levels of proteotoxic stress [27,28,41,42]. We did not, however, observe decreases in HSC70/HSP70 proteins on translating ribosomes in ALL cells, but rather increased association of chaperones to polysomes. This finding raises the possibility of VLX1570-induced effects on protein folding or associated processes, leading to decreased translational elongation. Inhibition of protein translation may contribute to the cytotoxicity of VLX1570 and may explain the limited level of resistance that develops during long-term culture in the presence of the compound [49].

Protein synthesis inhibition is a validated target for anticancer drugs [50]. Omacetaxine mepesuccinate is a semisynthetic analog of homoharringtonine that has been approved for treatment of chronic myeloid leukemia (CML) [51]. The drug disrupts the positioning of aminoacyl-tRNAs at the ribosome, thereby preventing translational elongation [52]. Translational inhibition is likely to contribute to the anticancer activity of VLX1570, but whether this effect will be at the expense of increased cytotoxicity to normal cells is not known at present. We found that protein synthesis in peripheral blood cells was affected by VLX1570. Previous studies reported limited toxicity of the VLX1570 analogue b-AP15 to peripheral blood cells [21], however, suggesting lower sensitivity to the effects of decreased protein synthesis. Here, we found that VLX1570 did not display developmental toxicity in zebrafish during the first five days of embryonal development at a concentration (1 µM) that reduced proliferation of RS4;11 cells. We are hopeful with regard to the possibility to develop suitable formulations of VLX1570, or soluble prodrugs, for future cancer therapy.

## 4. Materials and Methods

### 4.1. Reagents

Antibodies were from the following sources: ubiquitin Lys48 (#05-1307, Merck, Kenilworth, NJ USA), eIF2α (#5324S, Cell Signaling, Danvers, MA, USA), phospho-eIF2α (#9722S, Cell Signaling, Danvers, MA, USA ), CHOP (#5554 Cell Signaling, Danvers, MA, USA ), active caspase-3 (#9661S, Cell Signaling, Danvers, MA, USA ), BIP (#3183S, Cell Signaling, Danvers, MA, USA ), PARP #556494, BD Biosciences, San Jose, CA, USA), and β-actin (#A5316, Sigma-Aldrich, St Louis, MO, USA). Anti-HSC70/HSP70 antibody (clone N27), recognizing both HSC70 and HSP70, was purchased from Enzo Life Sciences, (Farmingdale, NY, USA) and anti-RPL10A (NBP2-47298). antibody was from Novus Biologicals, Centennial, CO, USA. 

### 4.2. Cell Culture

The human ALL cell lines MOLT-4, T-ALL, RS4:11, and SUP-B15 were obtained from the American Type Culture Collection (Manassas, VA, USA). Cells were gown as suspension in RPMI media supplemented with penicillin streptomycin and 10% FBS (Invitrogen, Carlsbad CA, USA). Viability was determined following exposure to compounds or DMSO using the MTT assay according to instructions by the vendor (Promega, Madison, WI, USA).

### 4.3. Protein Synthesis

Protein synthesis was measured by incorporation of Leucine L-(4,5-^3^H) (obtained from Perkin Elmer (NET1166005MC, Waltham, MA, USA). Cells were treated for drugs as indicated for 5 h and washed with PBS and RPMI-1640 media and 3.5 μCi/mL Leucine L-(4,5-^3^H) was added for 1 h. Cell suspensions were placed on Whatman GF/C microfiber filters and proteins were precipitated with ice-cold 5% trichloroacetic acid (TCA) (Sigma-Aldrich, St Louis, MO, USA). Filters were washed twice with 5% TCA, once with 99% ethanol, and left to dry. Radioactivity was measured using liquid scintillation counting.

### 4.4. Immunoblotting

Cell extract proteins were resolved by Tris-Acetate PAGE and Bis-Tris gels (Invitrogen, Carlsbad, CA, USA) and transferred onto polyvinylidene difluoride membranes, which were incubated overnight with primary antibodies, washed, and incubated with HRP-conjugated anti-rabbit Ig or anti-mouse Ig (Amersham Biosciences, Little Chalfont, UK) for 1 h.

### 4.5. Polysome Fractionation

Ribosome/polysomes were fractionated using 5–50% sucrose gradients as described in the video recording by Gandin et al. [39]. Cycloheximide was added to a concentration of 0.1 mg/mL 10 min prior to harvesting and was present in all buffers if not indicated otherwise. A Biocomp gradient station (BioComp Instruments, Fredericton, NB, Canada) was used for forming and collecting gradients. The system provides a high-resolution digital output, which was transferred to Adobe Illustrator for plotting of profiles. Proteins were concentrated by acetone precipitation from the gradient fractions and analyzed by immunoblotting.

### 4.6. Cell Labelling

RS4:11 cells were labeled with 1,1′-dioctadecyl-3,3,3′3´-tetramethylindocarbocyanine (DiI) as previously described [53]. Briefly, 70–80% confluent RS4:11 cells were washed with DPBS, covered with DiI at a final concentration of 4 µg/mL and incubated for 30 min at 37 °C. After labeling, cells were washed twice in DPBS and kept on ice prior to implantation in zebrafish embryos.

### 4.7. Zebrafish Assay

Transgenic Tg(fli1:EGFP)^y1^ zebrafish embryos [54] were obtained by natural breeding of adult fish (ZIRC, Eugene, OR, USA) and raised in E3-medium supplemented with PTU. ALL cells were resuspended at approximately 10^8^ cells per mL in cell growth medium and approximately 400 cells in 4 nL were implanted in the perivitelline space through sharp glass needles (world precision instruments, pulled in a PC-10 needle puller, Narishige, Tokyo, Japan) using a microinjection setup (MINJ-D, TriTech Research, Los Angeles, CA USA). Following injection, embryos were sorted for specific implantation of tumor cells in the perivitelline space and absence of cells in circulation under a fluorescent microscope (SMZ1500, Nikon, Tokyo Japan) and placed in E3 embryo medium containing 0.2 mM PTU. Three days following tumor implantation, the embryos were anesthetized in MS-222 (0.04%, Sigma-Aldrich, St. Lous, MO USA), and primary tumor sizes as well as the extent of local and peripheral, hematologous dissemination/metastasis of tumor cells were visualized under the fluorescent microscope. Results are shown as the average ± standard error of the mean of tumor volumes or number of cells present posterior to the anal opening. All animal experiments were approved by Linköpings Djurförsöksetiska Nämnd.

## Supplementary Materials

Supplementary Materials can be found at https://www.mdpi.com/1422-0067/21/13/4757/s1.

## Abbreviations

ALL	Acute lymphoblastic leukemia
ATF4	Activating Transcription Factor 4
CHOP	C/EBP homologous protein
CML	Chronic myeloid leukemia
CHX	Cyclohexamide
DiI	1,1′-dioctadecyl-3,3,3′3´-tetramethylindocarbocyanine
DUB	Deubiquitinase
ER	Endoplasmic reticulum
eIF2α	Eukaryotic initiation factor 2α
eIF2B	Eukaryotic translation initiation factor 2 subunit beta
HRI	Heme-related eIF2α kinase
HSP	Heat shock protein
ISR	Integrated stress response
ISRIB	Integrated Stress Response Inhibitor
mTOR	Mammalian target of rapamycin
PARP	Poly (ADP-ribose) polymerase
PERK	Protein kinase R (PKR)-like endoplasmic reticulum kinase)
TCA	Trichloroacetic acid
UPR	Unfolded Protein Response
