# Supplementary material for: Induction of ER Stress in Acute Lymphoblastic Leukemia Cells by the Deubiquitinase Inhibitor VLX1570
Source: Int J Mol Sci. 2020 Jul 4;21(13):4757. doi: 10.3390/ijms21134757 (PMC7369842; doi:10.3390/ijms21134757)
Supplement: Supplementary file 1 [file ijms-21-04757-s001.zip › Suppl Fig. 1.pdf]

Suppl Fig. 1

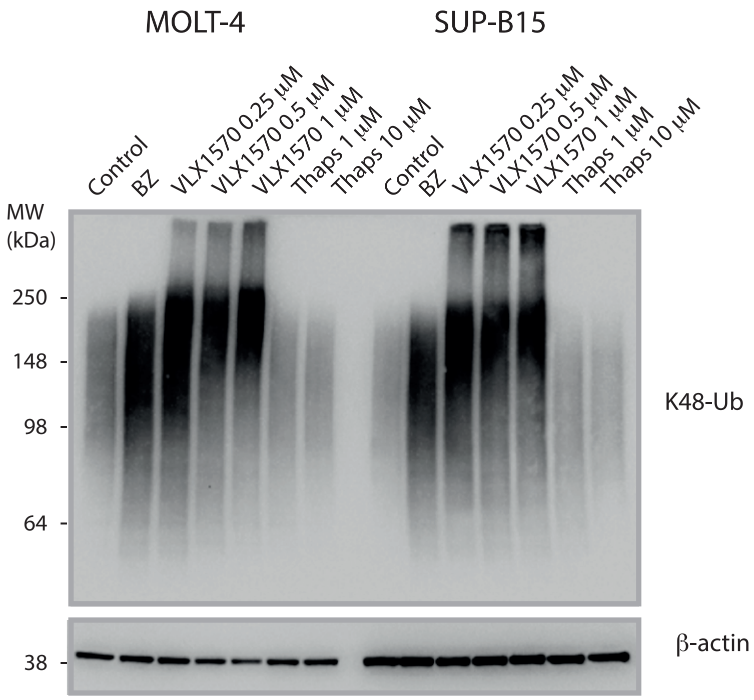

MOLT-4 and SUP-B15 cells were exposed to bortezomib (BZ; 50 nM), VLX1570, thapsigargin (10 μM) or vehicle (0.5% DMSO) for 6 h and extracts were prepared and subjected to immunoblotting.

.
