# Supplementary material for: Induction of ER Stress in Acute Lymphoblastic Leukemia Cells by the Deubiquitinase Inhibitor VLX1570
Source: Int J Mol Sci. 2020 Jul 4;21(13):4757. doi: 10.3390/ijms21134757 (PMC7369842; doi:10.3390/ijms21134757)
Supplement: Supplementary file 1 [file ijms-21-04757-s001.zip › Suppl Fig. 3.pdf]

Suppl. Fig. 3

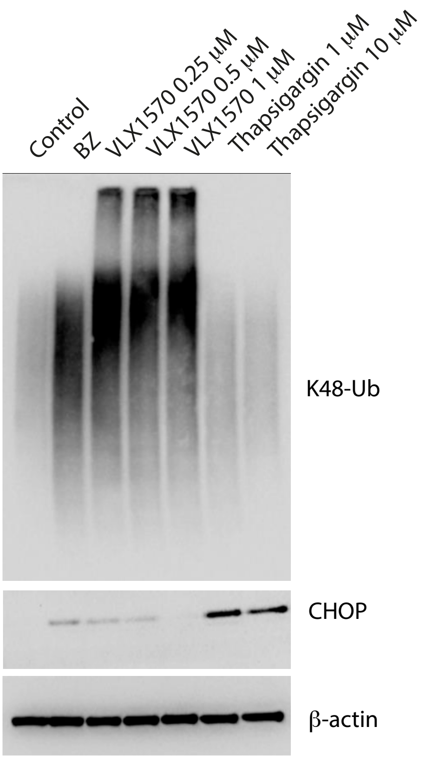

Induction of CHOP expression in SUP-B15 cells by thapsigargin. Cells were exposed to the indicated drugs for 9 hours. Lysates were prepared for immunoblotting using the indicated antibodies.
