# Supplementary material for: Induction of ER Stress in Acute Lymphoblastic Leukemia Cells by the Deubiquitinase Inhibitor VLX1570
Source: Int J Mol Sci. 2020 Jul 4;21(13):4757. doi: 10.3390/ijms21134757 (PMC7369842; doi:10.3390/ijms21134757)
Supplement: Supplementary file 1 [file ijms-21-04757-s001.zip › Suppl Fig. 4.pdf]

Suppl. Fig 4.

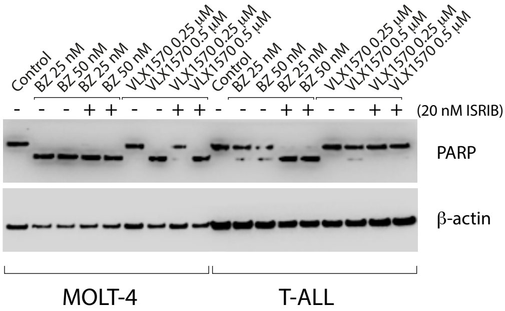

Drug-induced PARP cleavage at 12 hours of exposure. Note PARP cleavage also in T-ALL cells at this time point. Also note the strong stimulation of PARP cleavage by ISRIB in the T-ALL cell line.
