# Supplementary figures and images for: Induction of ER Stress in Acute Lymphoblastic Leukemia Cells by the Deubiquitinase Inhibitor VLX1570
Source: Int J Mol Sci. 2020 Jul 4;21(13):4757. doi: 10.3390/ijms21134757 (PMC7369842; doi:10.3390/ijms21134757)

Suppl. Fig. 2

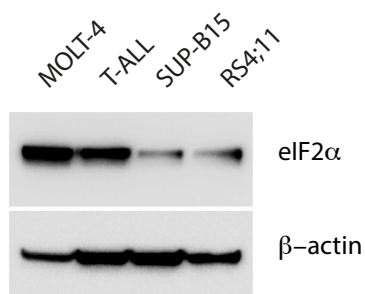

Basal levels of eIF2α in ALL cell lines.

Supplement: Supplementary file 1 [file ijms-21-04757-s001.zip › Suppl Fig. 2.pdf]

Suppl Fig. 5

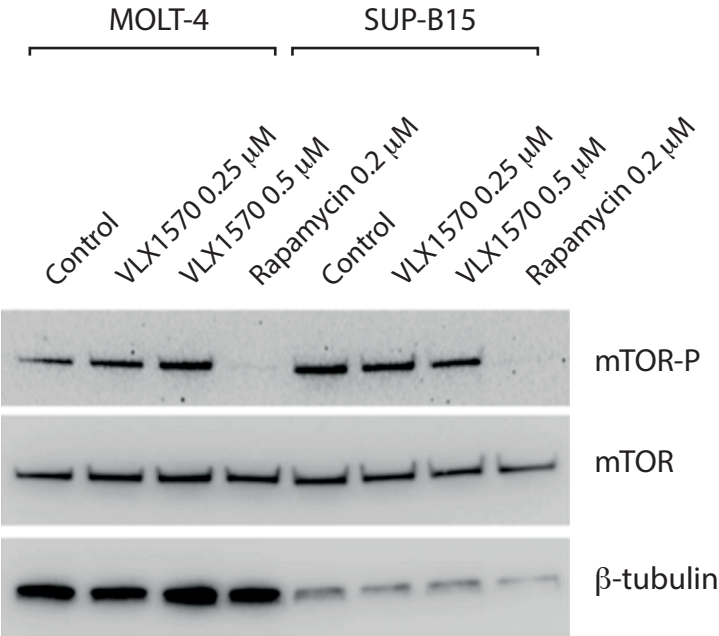

Supplement: Supplementary file 1 [file ijms-21-04757-s001.zip › Suppl Fig. 5.pdf]
